# Variation in Carbohydrates between Cancer and Normal Cell Membranes Revealed by Super‐Resolution Fluorescence Imaging

**DOI:** 10.1002/advs.201600270

**Published:** 2016-09-20

**Authors:** Junling Chen, Tianzhou Liu, Jing Gao, Lan Gao, Lulu Zhou, Mingjun Cai, Yan Shi, Wenyong Xiong, Junguang Jiang, Ti Tong, Hongda Wang

**Affiliations:** ^1^ State Key Laboratory of Electroanalytical ChemistryChangchun Institute of Applied ChemistryChinese Academy of SciencesChangchunJilin130022P. R. China; ^2^ The second hospital of Jilin universityChangchunJilin130022P. R. China; ^3^ Kunming institute of botanyChinese Academy of SciencesKunmingYunnan650201P. R. China; ^4^ University of Chinese Academy of SciencesBeijing100049P. R. China

**Keywords:** cancer markers, carbohydrates, distribution, dSTROM imaging

## Abstract

Carbohydrate alterations on cell membranes are associated with various cancer processes, including tumorigenesis, malignant transformation, and tumor dissemination. However, variations in the distributions of cancer‐associated carbohydrates are unclear at the molecular level. Herein, direct stochastic optical reconstruction microscopy is used to reveal that seven major types of carbohydrates tended to form obvious clusters on cancer cell membranes compared with normal cell membranes (both cultured and primary cells), and most types of carbohydrates present a similar distributed characteristic on various cancer cells (e.g., HeLa and Os‐Rc‐2 cells). Significantly, sialic acid is found to distribute in larger‐sized clusters with a higher cluster coverage percentage on various cancer cells than normal cells. These findings on the aberrant distributions of cancer‐associated carbohydrates can potentially serve as novel diagnostic and therapeutic targets, as well as making a contribution to clarify how abnormal glycosylations of membrane glycoconjugates participate in tumorigenesis and metastasis.

## 1 Introduction

Glycosylation is a common post‐translational modification that adds carbohydrates to membrane proteins and lipids. The resulting “glycoproteins” or “glycolipids” contain abundant carbohydrates on the extracellular side of the cell membranes and play integral roles in cell–cell and cell–matrix interactions by regulating cell adhesion, cell trafficking, and signal transduction.^1^, ^2^, ^3^ Abnormal glycosylation is associated with severe diseases, including tumorigenesis, malignant differentiation, and cancer metastasis and development.^4^, ^5^, ^6^

Carbohydrates on the cell surface have been recognized as modulators of interactions between cancer cells and their surrounding environment or other cells. As an intricate and ubiquitous process, glycosylation occurs on most proteins in mammalian cells. Thus, previous studies have primarily focused on a limited type of glycosylation events that impart tumor cell properties. Some common alterations of glycosylation have shown to generally accompany with tumorigenesis and metastasis, such as, increase in branches of N‐linked oligoaccharides,^7^, ^8^ changed sialylation on certain proteins or antigens,^5^, ^9^, ^10^ abnormal biosynthesis of O‐linked oligosaccharides.^11^, ^12^ Owing to the complexity and variability of carbohydrate structures, the cancer‐associated carbohydrate chains have no general changed feature. Determining ensemble changes in cancer‐related glycoconjugates is extremely challenging due to the vast variations in the glycoconjugates involved in cancer progression. Although the amount and type of carbohydrate chains are inconceivably huge, the type of elementary carbohydrate residues constituting diverse carbohydrate‐chains is fortunately limited, especially these in mammalian cells. Based on the core structures of N‐linked and O‐linked oligosaccharides,^13^ the common monosaccharide residues are the following ones:^13^ N‐acetyl‐D‐glucosamine (GlcNAc), N‐acetylgalactosamine (GalNAc), fucose (Fuc), mannose (Man), galactose (Gal), and sialic acid (Sia). Moreover, based on our related studies on carbohydrates6,^14^, ^15^, ^16^ we found the carbohydrates tended to form clusters as functional domains, where various functional glycocongujates locate via being cross‐linked by carbohydrate‐binding proteins (CBPs), with the contributions of lipid rafts as the stable factors and actin cytoskeletion as restricted factors. These findings indicate that the organization of carbohydrates not only represents the distributions of membrane glycoconjugates, but also is related to the membrane functional domains and membrane cytoskeletion. Herein, in addition to common monosaccharides mentioned above, we also selected one common oligosaccharide (pentasaccharide sequence Galβ1‐4GlcNAcβ1‐2 (Galβ1‐4GlcNAcβ1‐6) Manα1‐R) as the investigated target, which is a complement and comparison to monosaccharides, as well as confirms that the results of monosaccharides can reflect ones of carbohydrate‐chains. Through using super‐resolution microscopy with a nanometer‐level resolution,^17^, ^18^, ^19^ we studied the changed distributions of these selected carbohydrates on cell membranes, showing an overall alterant distribution of carbohydrate‐related molecules (glycoproteins, glycolipids, and glycosaminoglycans) containing the same carbohydrate residues, as well as suggesting the changed organization of cell membrane. Herein, we revealed the distributed alterations of carbohydrates on cancer cell (both cultured and primary cells) membranes compared with normal cell (both cultured and primary cells) membranes. Additionally, we also applied direct stochastic optical reconstruction microscopy (dSTORM) imaging to locate the distribution of Sia on multiple types of normal and cancer cells to confirm changed distributions of Sia is a common feature of various cells. Our findings on unusual organizations of cancer‐associated carbohydrates may provide complementary markers for the diagnosis of neoplastic disease and pave the way for elucidating how abnormal carbohydrates participate in cancer progression and metastasis.

## 2 Results and Discussion

### 2.1 Variations in Carbohydrate Distribution on Cancer and Normal Cell Membranes

**Figure 1:**
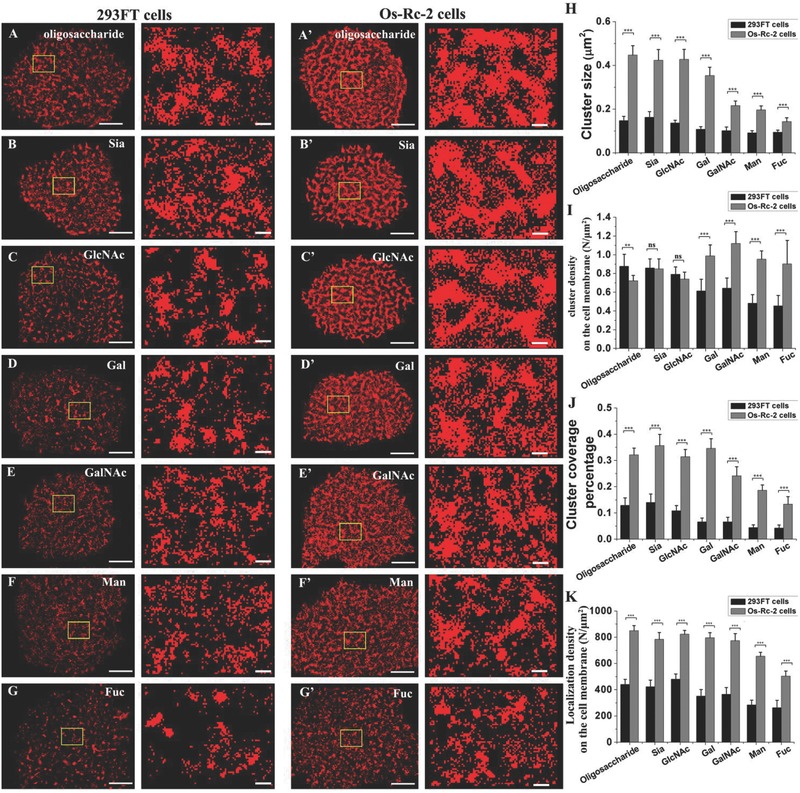
Characterization of the distribution of several representative carbohydrates on 293FT and Os‐Rc‐2 cell membranes by dSTORM imaging. A–G) The dSTORM images of seven types of carbohydrates on the 293FT cell membranes: A) oligosaccharide, B) Sia, C) GlcNAc, D) Gal, E) GalNAc, F) Man, and G) Fuc. A′–G′) The corresponding dSTORM images of the Os‐Rc‐2 cell membranes. H–K) The comparative statistical analyses of H) carbohydrate clusters based on cluster size, I) cluster density on the cell membrane, J) cluster coverage percentage, and K) localization density on the cell membrane. The scale bars represent 5 μm on the left sides of A–G and A′–G′ and 500 nm on the right sides of A–G and A′–G′. All results obtained from ten cell samples in three independent experiments are the means± S.D. and statistical significance by the two‐tailed unpaired t‐test is given with **P < 0.01; ***P < 0.0001; ^ns^ *P* > 0.05.

To ensure that the carbohydrates on the Os‐Rc‐2 (human renal carcinoma cell line) cell membranes were adequately labeled, we performed dSTORM imaging to localize the carbohydrates of interest under increasing labeling concentrations of their specific Alexa Fluor 647‐conjugated lectins. We determined the saturated labeling concentrations for all carbohydrates of interest based on their concentration gradient curves (Figure S1, in the Supporting Information). Then, we acquired nanoscaled morphologies of the carbohydrates of interest on the 293FT (human embryonic kidney cell) (**Figure** **1**A–G) and Os‐Rc‐2 cell membranes (Figure 1A′–G′) using the same labeling concentration (the saturating labeling concentration determined for Os‐Rc‐2 cells) through dSTORM imaging. All types of carbohydrates present significant differences in their distributions between the 293FT and Os‐Rc‐2 cells. The enlarged images show that these carbohydrates are primarily aggregated into larger clusters on the Os‐Rc‐2 cell membranes than on the 293FT cell membranes. The representative images of clusters abstracted from the reconstructed dSTORM images clearly display detailed patterns of carbohydrate clusters (Figure S2, in the Supporting Information). Using statistical analysis, we found that the significant differences in the carbohydrate distributions included cluster size, cluster coverage percentage, and localization density on the cell membrane. However, not all examined carbohydrates exhibit altered parameters, for instance, few to no changes in cluster densities are observed for oligosaccharide, Sia and GlcNAc. In detail, Gal, GlcNAc, oligosaccharide and Sia exhibit relatively high increases in cluster size (≈229%, ≈214%, ≈205%, and ≈161%, respectively, Figure 1H), compared with the cluster sizes on the 293FT cell membranes, suggesting that the carbohydrates on cancer cells are more prone to cluster formation. The cluster densities of Fuc, Man, GalNAc, and Gal show relatively high increases on the Os‐Rc‐2 cells (≈104%, ≈97%, ≈74%, and ≈61%, respectively, Figure 1I), indicating that more clusters of these carbohydrates are newly generated on cancer cells. By contrast, oligosaccharide, GlcNAc, and Sia are slightly reduced (≈10%, ≈6%, and ≈1%, respectively, Figure 1I), demonstrating that these carbohydrates prefer to aggregate into larger clusters rather than create new clusters on cancer cell membranes. The cluster coverage percentages of all carbohydrates are elevated more than onefold (Figure 1J). Particularly, Gal, Man, and GalNAc are largely elevated by ≈427%, ≈320% and ≈267%, respectively, indicating that higher extents of cluster coverage occur on the cancer cell membranes than on the normal cell membranes. The increased localization density on the Os‐Rc‐2 cell membranes (Figure 1K) suggests that these carbohydrates are expressed at higher levels on cancer cells (especially Man, Gal, and GalNAc with ≈131%, ≈127% and ≈112%, respectively).

Taken together, our results indicate that cancer cell membranes contain distinct distribution patterns of all types of carbohydrates compared to normal cell membranes. As well as having higher expression levels of these carbohydrates, cancer cells are covered with larger degrees of carbohydrate cluster coverage by mainly aggregating into larger clusters (GlcNAc, oligosaccharide, and Sia) or mainly forming more neonatal clusters (Fuc, Man, and GalNAc), or both (Gal). Based on our previous study on carbohydrates, carbohydrate clusters are formed by crosslinking of CBPs (i.e., galectins). During tumor progression, not only expression of specific carbohydrates increased, but expression of endogenous lectins (i.e., galectins) has also been reported to raise.^20^, ^21^ Therefore, we infer that the more significant clustering of carbohydrate on cancer cell membranes, with representing a global reorganization of all cell surface glycoconjugates, is the result of stronger interactions of carbohydrate ligands and CBPs. In fact, the interactions of carbohydrates with CBPs can affect tumor cell interactions with normal cells or with the extracellular matrix during metastatic spread and growth. These bindings of carbohydrate ligands and endogenous lectins not only occur in the formation of carbohydrate patterns on cell membranes (i.e., via galectins on tumor cells),^21^ but also in interactions of tumor cells and local microenvironment (via selectins on the endothelial surface,^22^ or via siglecs on macrophages^23^) in various metastasis processes. As known, carbohydrate‐related molecular interactions are generally weak and of low affinity, thus multivalent bindings between CBPs and carbohydrates via clustering together carbohydrates ligands of glycoconjugates are reported to be typically necessary in various cellular processes.^24^, ^25^, ^26^ Polyvalent interactions generally occur throughout biology, with the following functional advantages:^27^ it can provide a strong binding of ligands with modest or low surface area; it provides a graduated (graded) response to a biological signal via generating a broad range of signal strengths; it is able to obtain a great strength and specificity by heteromeric polyvalency of a mixture of ligand–receptor pairs; it can also induce macroscopic reorganizations of molecules or conformation of one or both of the interacting species; it has an ability to multiply an existing interaction or constructe a new one for biological evolution; it also serves as natural polyvalent inhibitors to prevent undesired interaction. Thus, according to the detailed functions of changed glycosylations in various cancer processes, including decreasing contact inhibition and substratum adhesion,^28^ increasing immigration,^7^ promoting the homotypic aggregation of cancer cells and the docking of tumor cells to endothelial cells,^11^ as well as the functional advantages of polyvalent interactions, we infer that the carbohydrates on cancer cell membranes could be reorganized to form larger clusters or more new clusters via polyvalent interactions with endogenetic lectins (i.e., galectins). In this way, carbohydrate ligands concentrate into large cluster to increase the regional density, which makes themselves easier to participate in interactions with extracellular matrix, homologous or heterogeneous cells, with higher affinity and specificity for CBPs. Additionally, changing density of reacting carbohydrate ligands may be accompanied with altering size and orientation of carbohydrates and the dimensional relationship between ligands and CBPs,^25^, ^29^ which will affect the accessibility of the carbohydrate ligands and finally promote or suppress the related processes. Moreover, alterations in protein glycosylations have been reported to perturb the structure and function of glycoproteins by changing their oligomerization, turnover, conformation, and interactions with other molecules.^30^, ^31^, ^32^, ^33^ Some computational models have also predicted that bulky glycoproteins favor transmembrane receptor organization (e.g., carbohydrate‐mediated integrin clustering would facilitate the assembly of mature adhesion complexes and enhance growth factor signaling).^34^, ^35^ Here, we propose that alterations in cancer‐related glycosylations may result in reorganization of cell surface glycoproteins (and glycolipids) into new clusters or recruitment into preexisting clusters, thereby directly or indirectly impacting the sensitivity of the receptor system to stimulation by affecting appropriate oligomerization.

### 2.2 Sia Distribution on Primary Normal and Cancer Cells

**Figure 2:**
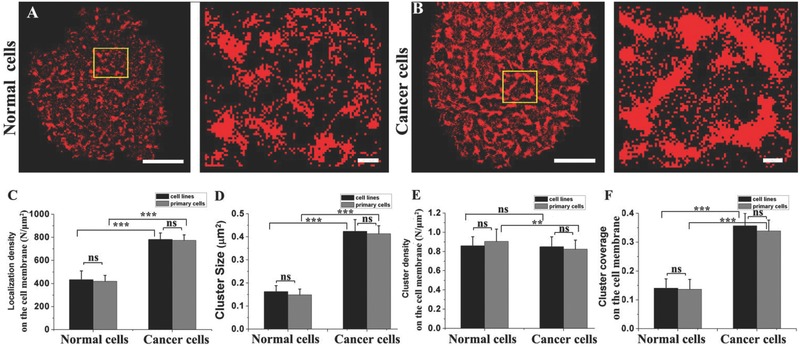
Sia distribution on primary normal kidney cells and kidney cancer cells revealed by dSTORM imaging. A,B) The distribution of Sia on (A) primary normal kidney cell and (B) kidney cancer cell membranes based on labeling with Alexa Fluor 647‐conjugated MAL. In A and B, the scale bars on the left represent 5 μm, and those on the right represent 500 nm. C–F) Histograms of the comparative analyses of distributed features between the cell lines and the primary cultured cells based on the localization density on the (C) cell membrane, (D) cluster size, (E) cluster density and (F) cluster coverage percentage. All results obtained from ten cell samples in three independent experiments are the means± S.D. and statistical significance by the two‐tailed unpaired t‐test is given with **P < 0.01; ***P < 0.0001; ns *P* > 0.05.

We further examined whether the distribution patterns of cancer‐associated carbohydrates were present in clinical specimens. Owing to the medium‐level variation in distribution among all examined carbohydrates, Sia was selected as a representative one for the study on primary normal kidney cells and kidney cancer cells. Compared with the normal kidney cells where Sias are primarily organized into a large number of small clusters with a relatively low expression level (**Figure** **2**A), the kidney cancer cells are densely covered with numerous large Sia clusters (Figure 2B). These results are similar to the results obtained from 293FT and Os‐Rc‐2 cells. Based on the statistical analysis, the abnormal morphology of Sia on kidney cancer cells is primarily due to changes in the localization density on the cell membrane (Figure 2C), cluster size (Figure 2D), and cluster coverage percentage (Figure 2F) but not the cluster density (Figure 2E). Importantly, the comparative analysis also reveals that there is almost no difference between the primary cells and the cultured cell lines, regardless of the distribution parameter, suggesting that the above results obtained from 293FT and Os‐Rc‐2 cells can be applied to the clinical samples. This finding indicates that the aberrant distribution of the carbohydrates of interest on cancer cells may be a valuable biomarker for the diagnosis of tumorigenesis.

### 2.3 The Similar Distributions of Carbohydrates between the Hela and Os‐Rc‐2 Cell Membranes

**Figure 3:**
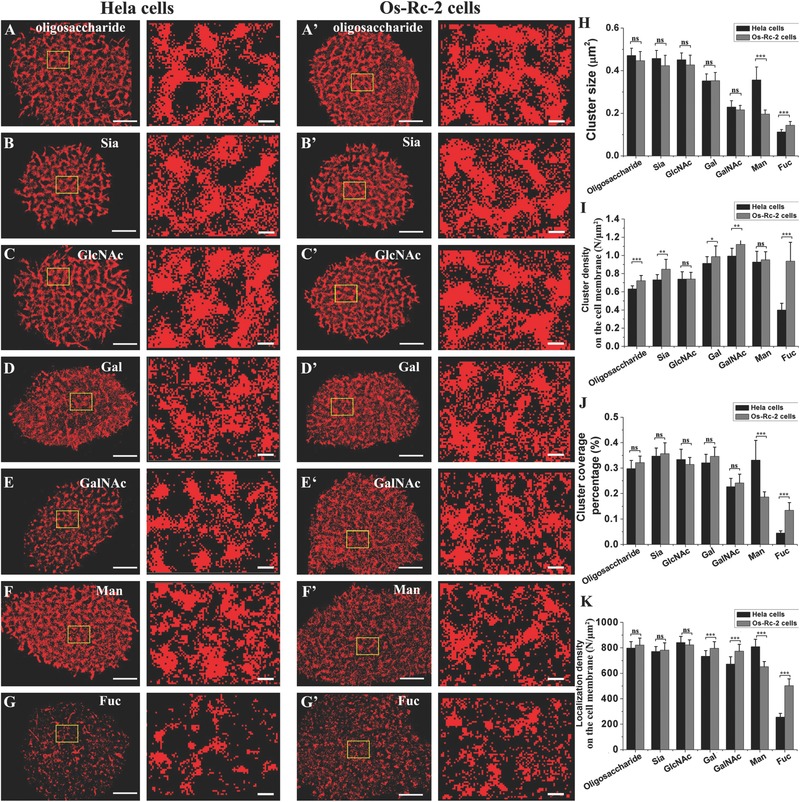
Cluster analyses of seven types of carbohydrates on different cancer cells. A–G) The distributions of seven major types of carbohydrates of interest on the Hela cell membranes. A) Oligosaccharide, B) Sia, C) GlcNAc, D) Gal, E) GalNAc, F) Man, and G) Fuc. A′–G′) The corresponding distributions of the carbohydrates on the Os‐Rc‐2 cell membranes. H–K) The systematic statistical analysis of the distribution characteristics of the carbohydrates of interest on the Hela and Os‐Rc‐2 cell membranes: H) cluster size, I) cluster density, J) cluster coverage percentage, and K) localization density on the cell membrane. In A–G and A′–G′, the scale bars on the left represent 5 μm, and those on the right represent 500 nm. All results obtained from ten cell samples in three independent experiments are the means± S.D. and statistical significance by the two‐tailed unpaired t‐test is given with *P < 0.05; **P < 0.01; ***P < 0.0001; ns *P* > 0.05.

Next, we systemically investigated the distribution patterns of the seven major types of carbohydrates on different cancer cells. To acquire the distributions of all carbohydrates of interest on the Hela (human cervical carcinoma cell line) cell membranes for comparison with the distributions on the Os‐Rc‐2 cell membranes, we determined the saturating concentrations of Alexa Fluor 647‐conjugated lectins needed to completely label all carbohydrates of interest on the Hela cells (Figure S3, in the Supporting Information), which was similar to the approach used with the Os‐Rc‐2 cells. Then, the distributions of all types of carbohydrates of interest on the Hela cell membranes (**Figure** **3**A–G) were imaged using their specific saturating labeling concentrations. Each type of carbohydrates is present in an apparent clustered state on the Hela cell membranes (Figure 3A–G), which was similar to the observations with the Os‐Rc‐2 cell membranes (Figure 3A′–G′). Extracting the clusters from the original reconstructed images allows the similar cluster distribution patterns to be more clearly displayed (Figure S4, in the Supporting Information). The statistical analysis reveals that the examined carbohydrates on the Hela cell membranes are distributed in clusters with sizes similar to the corresponding types of carbohydrate clusters on the Os‐Rc‐2 cells (Figure 3H), with the exception of Man and Fuc. By contrast, the cluster densities of the carbohydrates (with the exception of GlcNAc and Man) are altered on both cell lines (especially Fuc) (Figure 3I). Comparable cluster coverage percentages of carbohydrates occur on both cell lines, except for Man and Fuc (Figure 3J). Man and Fuc also have markedly different localization densities on different cell membranes, but the others have small (Gal and GalANc) or even no (oligosaccharide, Sia and GlcNAc) difference on the Hela and Os‐Rc‐2 cell membranes (Figure 3K). Taken together, the carbohydrates (except Man and Fuc) are distributed with similar patterns in cluster size, cluster coverage percentage, and localization density on both the Hela and Os‐Rc‐2 cancer cell membranes (especially oligosaccharide, Sia, and GlcNAc). These findings suggest that not only the incremental expression level of glycoconjugates but also the global alterations in distribution patterns (i.e., cluster size and cluster coverage percentage) may be unique distribution features of different cancer cells.

### 2.4 Comparative Analysis of Sia Distribution on Various Normal and Cancer Cell Membranes

**Figure 4:**
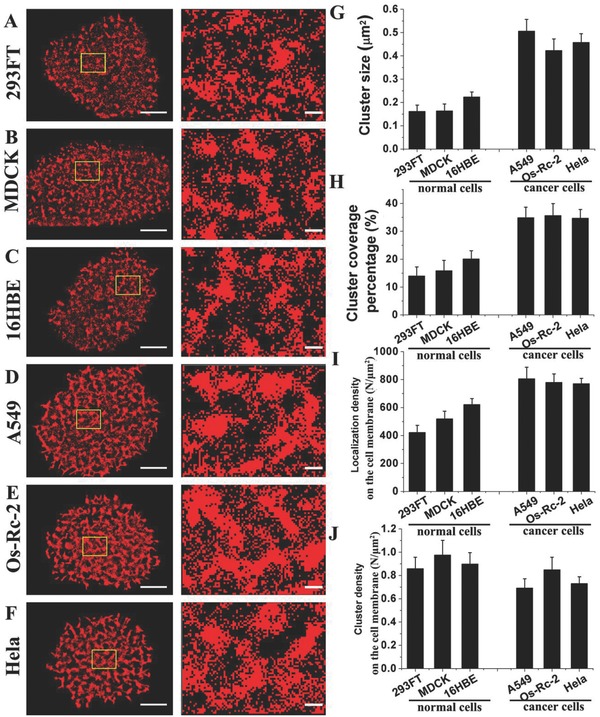
Analysis of Sia distribution on various normal and cancer cell membranes. A–F) The dSTORM reconstruction images (the left) and the corresponding enlarged images (the right) of Sia on several cell lines: (A) 293FT, (B) MDCK, (C) 16HBE, (D) A549, (E) Os‐Rc‐2, and (F) Hela. G–J) The histograms present (G) the cluster size, (H) cluster coverage percentage, (I) localization density on the cell membrane, and (J) cluster density on the cell membrane. In A–F, the scale bars on the left represent 5 μm, and those on the right represent 500 nm. All results obtained from ten cell samples in three independent experiments are the means ± S.D.

Based on the results obtained with the Hela and Os‐Rc‐2 cells, we hypothesized that the aberrant morphologies of some types of carbohydrates (oligosaccharide, Sia, GlcNAc, and Gal) might be a common feature of various cancer cell lines. Considering that Sia shows a medium‐level variation of distribution on the 293FT/Os‐Rc‐2 or Hela/Os‐Rc‐2 cell membranes, Sia was selected as a representative carbohydrate to investigate the distribution variations in various normal and cancer cell lines. Using the same labeling concentration determined with the Alexa Fluor 647‐conjugated Maackia amurensis lectin (MAL) (the saturating concentration for Os‐Rc‐2 cells), we investigated Sia morphology on MDCK (Madin‐Darby canine kidney cell), 16HBE (human bronchial epithelial cell), and A549 (human lung cancer cell line) cells. By systematically comparing the dSTORM images of Sia on various normal and cancer cell membranes (**Figure** **4**A–F), we easily distinguished the normal cell lines (293FT, MDCK, and 16HBE) from the cancer cell lines (A549, Os‐Rc‐2, and HeLa) based on the markedly different Sia distributions. Sias tend to form relatively small clusters on the normal cell lines. The average cluster sizes are ≈0.16 μm^2^ on 293FT cells, ≈0.16 μm^2^ on MDCK cells, and ≈0.22 μm^2^ on 16HBE cells (Figure 4G). However, Sias generally gather into large clusters on the cancer cell lines, with average cluster sizes of ≈0.51 μm^2^ on A549 cells, ≈0.42 μm^2^ on Os‐Rc‐2 cell, and ≈0.46 μm^2^ on the Hela cells (Figure 4G). Each normal cell line is covered with smaller percentages of clusters (≈14.0% on 293FT cells, ≈15.9% on MDCK cells, and ≈20.1% on 16HBE cells) than the corresponding cluster coverage on the cancer cell lines (≈34.9% on A549 cells, ≈35.6% on Os‐Rc‐2 cells, and ≈34.7% on Hela cells, Figure 4H). Interestingly, the Sia localization density differs significantly between the 293FT and Os‐Rc‐2 cells from the same tissue but not between the other normal and cancer cell lines from different tissues (Figure 4I), indicating that this considerable alteration in the Sia expression level is present only in the normal and cancer cells from the same tissue and thus failed as a general characteristic of the various cancer cells. The Sia cluster density varies irregularly between the various normal and cancer cell lines (Figure 4J), suggesting that the alterations in the Sia cluster density are not related to tumorigenesis. Overall, the significant differences in the carbohydrate distribution patterns based on Sia cluster size and coverage, rather than localization and cluster densities, may serve as markers for various neoplastic diseases. Indeed, each type of cells always expresses their specified proteins containing various carbohydrates, thus a general expression trend of carbohydrates cannot occur on multiple types of cancer cells. However, the distributed patterns of carbohydrates represent a global alteration in topography of cell membranes via integrating the organizations of membrane glycoconjugates, membrane functional domains, and membrane cytoskeleton, so that the changed distributions of carbohydrates have a capacity to be general features for various cancer cells.

## 3 Conclusion

Although alterations in complex O‐glycosylation, N‐glycosylation, and terminal carbohydrate structures were reported to most likely impact the survival and progression of neoplastic cells in some studies,^28^, ^36^, ^37^ no studies have investigated the detailed variations in the distribution of carbohydrates accompanied with tumorigenesis. Herein, based on the core structures of N‐linked and O‐linked oligosaccharides, we selected six common monosaccharides as the investigated targets. For a comprehensive study and confirming there is no significant difference between monosaccharides and oligosaccharides, we also selected one common pentasaccharide sequence as the examined carbohydrate. With dSTORM imaging, we systematically investigated the morphologies of seven major types of carbohydrates on various normal and cancer cell lines. These examined carbohydrates not only express at higher levels but also distribute in more distinct patterns (especially larger cluster sizes and higher cluster coverage percentages) on Os‐Rc‐2 cells (cancer cells) than on 293FT cells (normal cells), showing these distinct distributions of carbohydrates can potentially serve as markers of kidney cancer cells. Moreover, owing to a medium‐level distribution variation between the Os‐Rc‐2 and 293FT cells, Sia was selected as a representative carbohydrate to be examined on clinical specimens, suggesting that the significant alterations in the distributions of the examined carbohydrates have great potentialities to serve as novel clinical diagnostic indicators. Most types of examined carbohydrates (oligosaccharide, Sia, GlcNAc, Gal, and GalNAc) are found to distribute in similar patterns on Hela and Os‐Rc‐2 cells, indicating that these specified distribution patterns are likely a common feature of different cancer cells. Moreover, as Sia always exhibits a common behavior among all examined carbohydrates, it was still selected as the representative one for the study of various cancer cell lines. We found Sia could form similarly sized clusters and exhibit similar cluster coverage percentages, which is markedly distinct from that on various normal cell lines. However, the carbohydrate expression level shows little to no variation, thus some conventional analytical methods (i.e., mass spectrometry and traditional fluorescence microscopy) were not able to detect cancer‐associated alterations in carbohydrates. Our findings demonstrate that dSTORM imaging with the predominant resolution can examine alterations in cell surface carbohydrates on various cancer cells to better identify tumorigenesis based on their distinct distribution patterns (especially cluster sizes and cluster coverage percentages). Additionally, the abnormal morphologies of these cancer‐related carbohydrates originate from alterations in the global distribution of glycoconjugates on cancer cell membrane, indicating that cancer‐related changes may occur in the density of ligands, the accessibility of interacting molecules, the conformation, and oligomerization of functional proteins or lipids, and so on. As a consequence, a distinct membrane organization which is either less prevalent or absent in normal cells is probably formed for tumor progression and metastasis. Although these hypotheses need to be confirmed, our findings on the aberrant distribution of cancer‐associated carbohydrates provide new insights into cancer studies, with promoting a demonstration of the mechanisms underlying the changes in glycosylation that mediate tumor metastasis and invasion.

## 4 Experimental Section

*Cell Culture—Cell Lines*: The Os‐Rc‐2, 16HBE, Hela, 293FT, A549, and MDCK cell lines were purchased from the Shanghai Institute of Biological Sciences (Shanghai, China). Cells were maintained in a 5% CO_2_ environment at 37 °C in their respective media (Os‐Rc‐2 and 16HBE cells in RPMI 1640 medium, HeLa, 293FT and A549 cells in DMEM, and MDCK cells in MEM; all media were purchased from HyClone) supplemented with 10% fetal bovine serum (FBS, HyClone), 100 U mL^–1^ penicillin and 100 μg mL^–1^ streptomycin.

For dSTORM imaging, the cells were cultured on clean cover slips (22 mm × 22 mm, Fisher) in their media for ≈24–36 h to achieve ≈70% confluence.

*Cell Culture—Primary Cultured Cells from Clinical Human Specimens*: This study was approved by the ethics review board at the Second Hospital of Jilin University. The clinical specimens were collected from all patients after obtaining written informed consent. The specimens used for the dSTORM experiments were kidney cancer cells and normal kidney epithelial cells freshly isolated from kidney cancer specimens and paired pathologically with normal kidneys. First, the human clinical samples were rinsed with 1× phosphate‐buffered saline (PBS) six to eight times to remove impurities and blood cells. After removing excess tissues, such as necrotic cells and adipose, the samples were minced into small pieces (≈1 mm^3^) in 1 mL of DMEM‐F12 medium. After washing with PBS, the minced tissues were incubated with collagenase IV (Invitrogen, final concentration of 1 mg mL^–1^) at 37 °C for 100 min with shaking. The tissues in solution were passed through a 60–80 μm^2^ filter, and the cell suspension was centrifuged at 1000 rpm for 4 min. The supernatant was then discarded, and the cell pellet was resuspended in 1 × PBS with 3% FBS. These centrifugation and resuspension procedures were repeated three times. The cell solution was resuspended in DMEM‐F12 medium supplemented with 20% FBS, 100 U mL^–1^ penicillin and 100 μg mL^–1^ streptomycin, dropped onto a small glass slide in a culture dish at an appropriate cell density, and maintained in a 5% CO_2_ environment at 37 °C.

*Sample Preparation for dSTORM Imaging—Preparation of Alexa Fluor 647‐Conjugated Lectins*: Several common lectins were selected to recognize their specific carbohydrates according to their binding specificity (information provided by the manufacturer's introduction). Lectin from Phaseolus vulgaris (PHA‐L, from EY laboratories) for oligosaccharide,^38^, ^39^ Maackia amurensis lectin (MAL, from Sigma) for sialic acid linked to galactose by an α2‐3 linkage,^40^, ^41^ wheat germ agglutinin (WGA, from Sigma) mainly binding to GlcNAc and its β‐(1→4)‐linked oligosaccharides,^38^, ^42^ erythrina cristagalli lectin (ECL, from Sigma) for D‐Galβ1‐4GlcNAc (Gal),^38^ lectin from glycine max (soybean) (SBA) with high affinity for GalNAc,^42^, ^43^ lectin from Morniga M (MNA‐M, from EY laboratories) for Man,^44^ and lectin from Anguilla anguilla (eel) (AAA, from Sigma) for Fuc.^38^, ^45^ With finishing the reaction of the lectin solution with Alexa647 (Molecular Probes) at 27 °C for ≈3 h with gentle vortex, the redundant free Alexa647 was removed by using a PD Spin Trap G‐25 filtration column (GE Healthcare). Finally, according to the Beer‐Lambert, the labeling ratio was calculated law by measuring the absorbance of sample at 280 nm (lectin) and 650 nm (maximum absorbance of Alexa Fluor 647).

*Sample Preparation for dSTORM Imaging—For dSTORM Imaging of the Cell Lines*: The cultured cells were washed three times with 1× PBS and then fixed in 1 mL of 4% paraformaldehyde in 1× PBS at 37 °C for 40–60 min. After rinsing three times with PBS, the cells were stained with 50 μL of Alexa Fluor 647‐conjugated lectin solution at 4 °C in the dark for 10 min. Then, after removing the excess solution, the cells were washed four times with 1× PBS.

*Sample Preparation for dSTORM Imaging—For dSTORM Imaging of Primary Cultured Cells*: After fixation, the cultured cells were labeled with fluorescein isothiocyanate‐labeled antihuman CD326 (EpCAM, Biolegend, diluted 1:40 with PBS) at 4 °C in the dark for 20 min and washed four times with 1× PBS. Then, the cells were incubated with Alexa Fluor 647‐conjugated MAL at 4 °C in the dark for 10 min and washed four times with 1× PBS.

Before imaging, 30 μL of imaging buffer (containing 140 × 10^–3^ m beta‐mercaptoethanol, 0.5 mg mL^–1^ of glucose oxidase and 40 μg mL^–1^ of catalase) was dropped onto a microscope slide (24 mm × 50 mm, Fisher), and the small coverslip containing the seeded cells was gently sealed onto the large microscope slide using nail polish.

*dSTORM Imaging*: Using an inverted Nikon Ti‐E microscope with an oil‐immersion objective (100×, 1.49 NA, Nikon, Japan), dSTORM imaging was implemented under total internal reflection fluorescence illumination, which can significantly decrease the background noise around single molecules. During imaging, a 640 nm laser was responsible for Alexa Fluor 647 fluorescent excitation and fluorophore photoswitching. Additionally, an electron‐multiplying charge‐coupled device (EMCCD, Photometrics, Cascade II) camera was used to acquire frames (512 × 512 pixels) at high speed with a pixel size of 160 nm. Finally, 5000 frames with a 40 ms exposure time were collected using Micro‐Manager, based on ImageJ (U.S. National Institutes of Health), to reconstruct the dSTORM images with QuickPALM. During imaging, the sample was stabilized with two clips to reduce the possibility of x‐y drift, and z‐drift was eliminated with a focus lock. Due to the very short acquisition time, the stage shift could be ignored.

*Data Analysis*: Characterization of the carbohydrate cluster patterns was performed via image‐based cluster analysis. First, the outlines of the clusters became clearer after removing the sparse single points with the ‘denoise’ function in ImageJ. Then, the reconstructed image was converted to a binary image and extracted the qualified clusters by ‘Analysis Particles’ in ImageJ with setting the cluster area and the circularity. Finally, useful morphological information was acquired from the qualified clusters by setting the measurement in ImageJ, including the average area size, the circularity, the total number of clusters, and the total area of all clusters. The cluster density and the cluster coverage percentage on the cell membrane were calculated by further measuring the cell membrane area. Moreover, we used MATLAB to acquire an accurate total number of localization events on the cell membrane and to calculate the localization density on the cell membrane.

## Supporting information

As a service to our authors and readers, this journal provides supporting information supplied by the authors. Such materials are peer reviewed and may be re‐organized for online delivery, but are not copy‐edited or typeset. Technical support issues arising from supporting information (other than missing files) should be addressed to the authors.

Supplementary
