# Supplementary material for: Variation in Carbohydrates between Cancer and Normal Cell Membranes Revealed by Super‐Resolution Fluorescence Imaging
Source: Adv Sci (Weinh). 2016 Sep 20;3(12):1600270. doi: 10.1002/advs.201600270 (PMC5157168; doi:10.1002/advs.201600270)
Supplement: Supplementary file 1 — Supplementary [file ADVS-3-0-s001.pdf]

Copyright WILEY-VCH Verlag GmbH & Co. KGaA, 69469 Weinheim, Germany, 2016.

ADVANCED SCIENCE
Open Access

Supporting Information

for *Adv. Sci.*, DOI: 10.1002/advs.201600270

Variation in Carbohydrates between Cancer and Normal Cell Membranes Revealed by Super-Resolution Fluorescence Imaging

Junling Chen, Tianzhou Liu, Jing Gao, Lan Gao, Lulu Zhou, Mingjun Cai, Yan Shi, Wenyong Xiong, Junguang Jiang, Ti Tong, and Hongda Wang*

Supporting information

Variation in carbohydrates between cancer and normal cell membranes revealed by super-resolution fluorescence imaging

Junling Chen,[a,b] *Tianzhou liu,*[c] *Lan Gao,*[b,d] *Lulu Zhou,*[a,b] *Mingjun Cai,*[a] *Yan Shi,*[a] *Junguang Jiang,*[a] *Ti Tong**[,c] *and Hongda Wang**[, a]

Dr. J. Chen, Dr. L Zhou, M. Cai, Dr. Y. Shi, Dr. J. Jiang, Prof. H.Wang, State Key Laboratory of Electroanalytical Chemistry, Changchun Institute of Applied Chemistry, Chinese Academy of Sciences, Changchun, Jilin 130022, P.R. China. E-mail: hdwang@ciac.ac.cn; tti666@163.com

Dr. J. Chen, Dr. L. Gao, Dr. L Zhou, University of Chinese Academy of Sciences, Beijing 100049, P.R. China.

Dr. T. Liu, Prof. T. Tong, The second hospital of Jilin university, Changchun, Jilin 130022, P.R. China. E-mail: tti666@163.com

Dr. L. Gao, Kunming institute of botany, Chinese Academy of Sciences, Kunming, Yunnan 650201, P.R. China.

1. Determination of the saturating concentrations of Alexa Fluor 647-conjugated lectins required to labels pecific carbohydrates on Os-Rc-2 cell membranes.

To ensure that each type of carbohydrate of interest was adequately labeled, we localized carbohydrate distributions using increasing labeling concentrations of specific Alexa Fluor 647-conjugated lectins. Then, we determined the saturating labeling concentration for each type of carbohydrate based on the concentration gradients acquired by calculating the localization densities on the cell membrane at different labeling concentrations (Fig. S1). The saturating concentrations were ~3 μg/mL of *Maackia amurensis lectin* (MAL) for sialic acid (Sia), ~2.4 μg/mL of *Wheat-germ agglutinin* (WGA) for N-acetylglucosamine (GlcNAc), ~3.5 μg/mL of *Erythrina cristagalli lectin* (ECL) for galactose (Gal), ~4.2 μg/mL of the lectin from *Phaseolus vulgaris* (PHA-L) for oligosaccharide, ~3.5 μg/mL of the lectin from *Morniga M* (MNA-M) for mannose (Man), ~7 μg/mL of *Soy bean agglutinin* (SBA) for N-acetylgalactosamine (GalNAc), and ~14.5 μg/mL of the lectin from *Anguilla anguilla* (eel) (AAA) for fucose (Fuc).

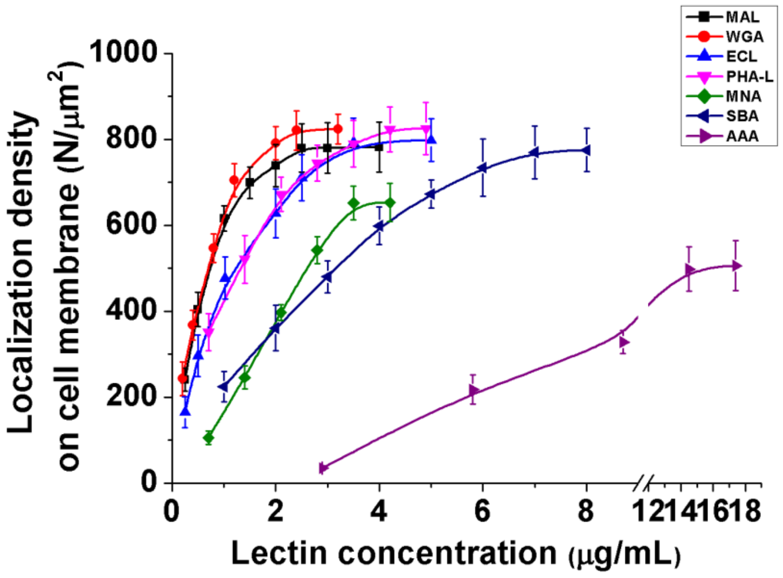

Figure S1. The concentration gradient curves of the seven types of carbohydrates of interest on Os-Rc-2 cell membranes. We used MAL for Sia (in black), WGA for GlcNAc (in red), ECL for Gal (in blue), PHA-L for oligosaccharide (in pink), lectin

MNA-M for Man (in green), SBA for GalNAc (in dark blue), and AAA for Fuc (in purple).

2. Distributions of the qualified clusters of the seven types of carbohydrates on 293FT and Os-Rc-2 cell membranes.

After image-based cluster analysis with ImageJ, we acquired detailed carbohydrate cluster morphologies. The qualified clusters were abstracted from the original reconstructed dSTORM images, along with detailed information on the cluster, including the average cluster size, the total number of clusters, and the total cluster area, by setting a cluster size threshold. Without single points, the distribution pattern of each carbohydrate cluster became more distinctive (Fig. S2). All types of carbohydrates formed numerous small clusters on the 293FT membranes (Fig. S2A-G-upper); however, these carbohydrates were differentially distributed on the Os-Rc-2 membranes and exhibited stronger clustering capabilities (Fig. S2A-G-lower).

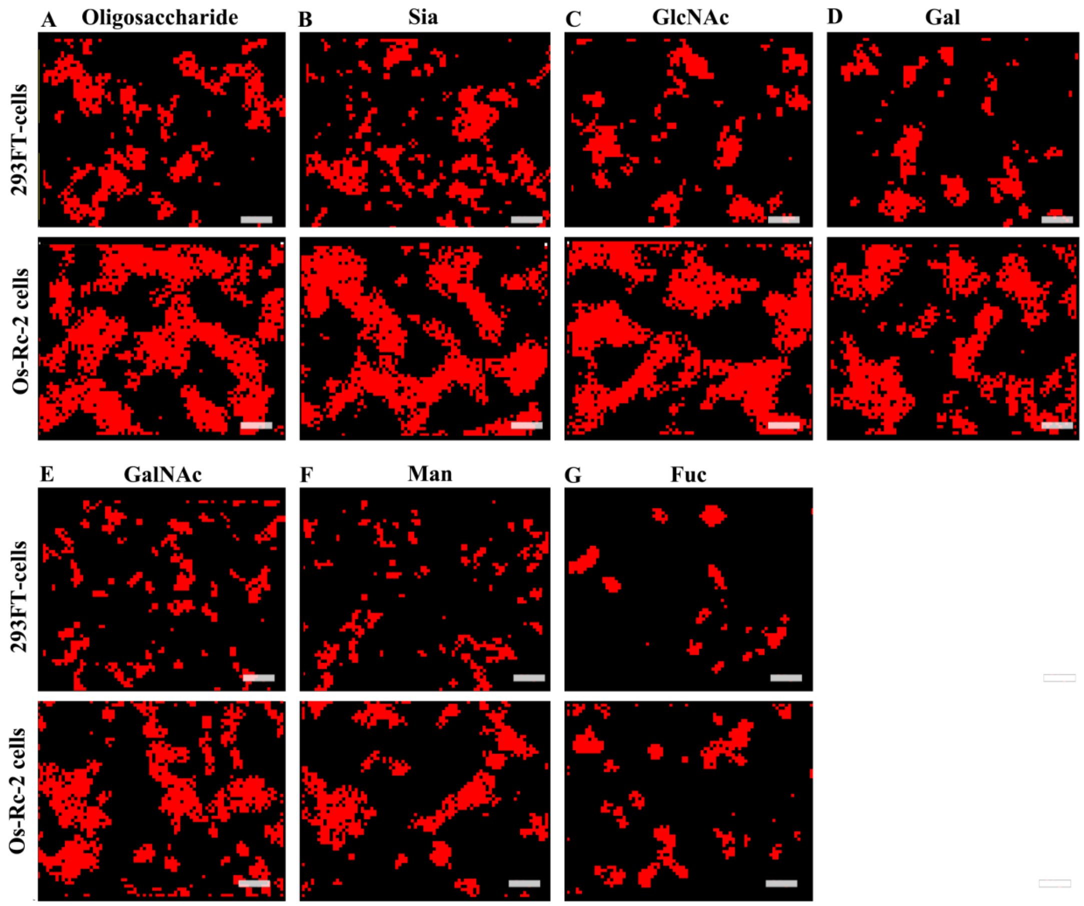

Figure S2. The distributions of the qualified carbohydrate clusters on 293FT (upper) and Os-Rc-2 cell membranes (lower): oligosaccharide (A), Sia (B), GlcNAc (C), Gal (D), GalNAc (E), Man (F), and Fuc (G). The scale bars in A-G represent 500 nm.

3. Determination of the saturating concentrations of Alexa Fluor 647-conjugated lectins used to label specific carbohydrates on HeLa cell membranes.

To examine whether the aberrant distribution of carbohydrates observed on Os-Rc-2 cellswas a common characteristic of other cancer cells, we selected HeLa cells as a representative cell line for comparison with the Os-Rc-2 cell line. Similar to the determination of the saturating labeling concentration in Os-Rc-2 cells, we acquired the saturating concentrations of Alexa Fluor 647-conjugated lectins in HeLa cells based on the concentration gradient curves (Fig. S3): ~3 μg/mL of MAL for Sia, ~4.5

μg/mL of WGA for GlcNAc, ~3.5 μg/mL of ECL for Gal,~7.4 μg/mL of PHA-L for oligosaccharide, ~3.5 μg/mL of MNA-M for Man, ~14.9 μg/mL of SBA for GalNAc, and ~12.8 μg/mL of AAA for Fuc.

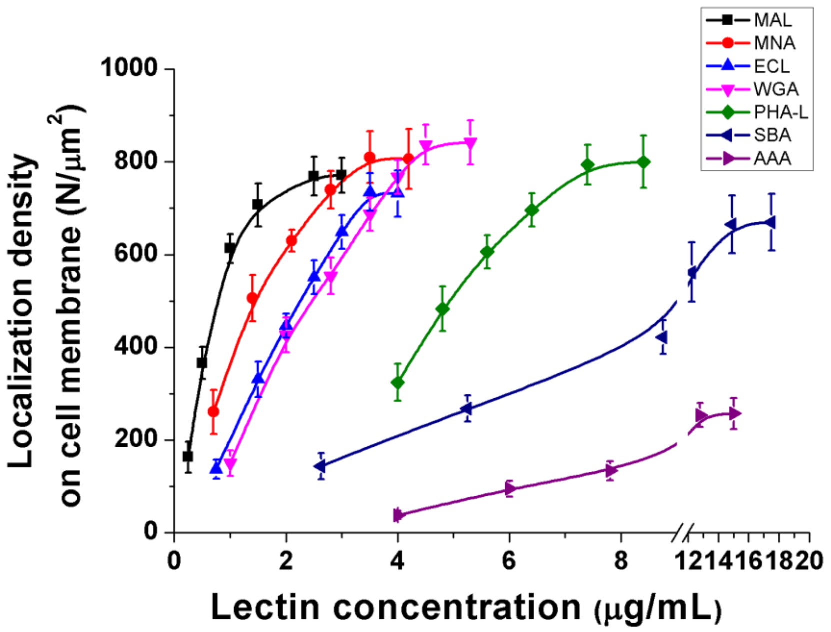

Figure S3. The concentration gradient curves of the seven types of carbohydrates of interest on HeLa cell membranes: MAL for Sia (in black), WGA for GlcNAc (in red), ECL for Gal (in blue), PHA-L for oligosaccharide (in pink), lectin MNA-M for Man (in green), SBA for GalNAc (in dark blue), and AAA for Fuc (in purple).

4. Distributions of the qualified clusters of the seven types of carbohydrates on HeLa and Os-Rc-2 cell membranes.

Using image-based cluster analysis, we acquired the distributions of the qualified carbohydrate clusters on the HeLa cell membranes (Fig. S4A-G-upper). Similar to the distributions of the carbohydrates of interest on the Os-Rc-2 cell membranes (Fig. S4A-G-lower), these carbohydrates were distributed with significant clustering characteristics. Based on visual observation, almost no differences were detected in the distribution patterns of the carbohydrate clusters between the cell lines, with the exception of Man.

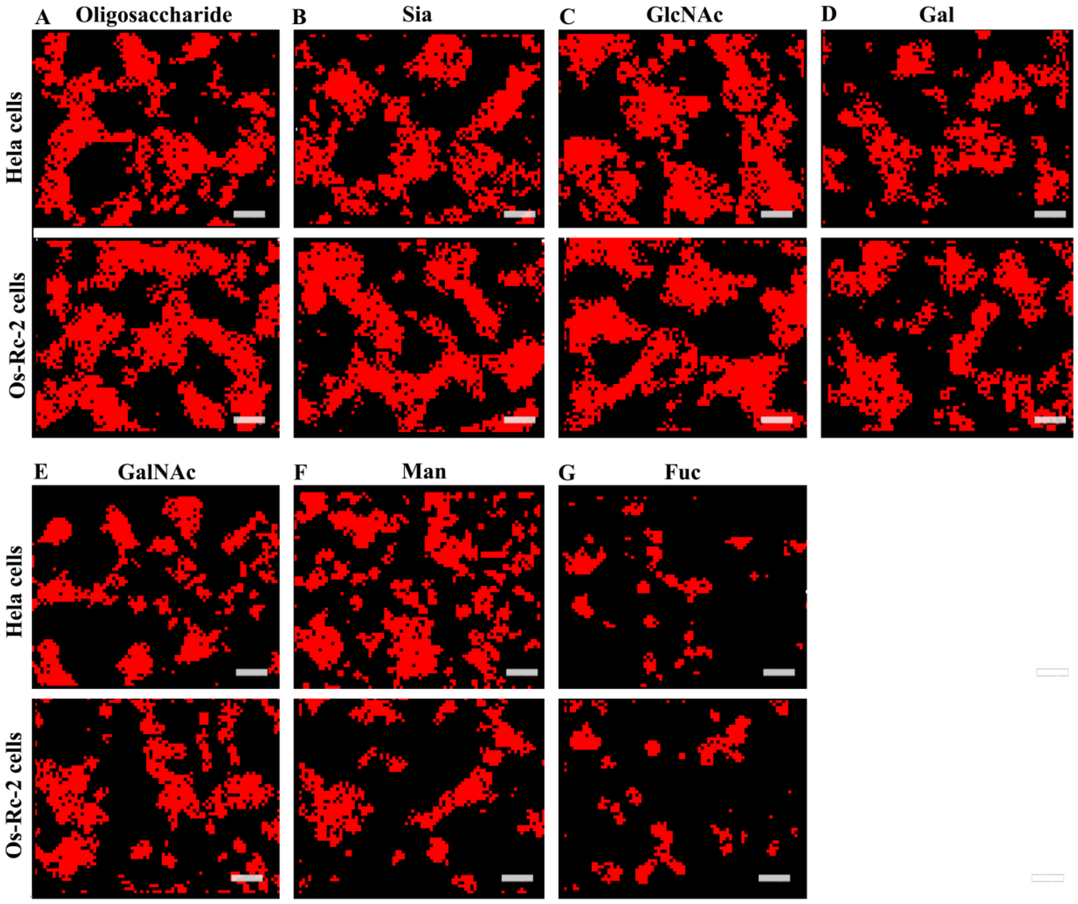

Figure S4. Distributions of the qualified carbohydrate clusters on HeLa (upper) and Os-Rc-2 cell membranes (lower): oligosaccharide (A), Sia (B), GlcNAc (C), Gal (D), GalNAc (E), Man (F), and Fuc (G). The scale bars in A-G represent 500 nm.
